# The Effects of Leader Emotional Intelligence, Leadership Styles, Organizational Commitment, and Trust on Job Performance in the Real Estate Brokerage Industry

**DOI:** 10.3389/fpsyg.2022.881725

**Published:** 2022-05-12

**Authors:** Chun-chang Lee, Yei-shian Li, Wen-chih Yeh, Zheng Yu

**Affiliations:** ^1^Department of Real Estate Management, National Pingtung University, Pingtung, Taiwan; ^2^Department of Real Estate Management, HungKuo Delin University of Technology, New Taipei City, Taiwan; ^3^Department of Land Economics, National Chengchi University, Taipei, Taiwan

**Keywords:** real estate brokers, leadership emotional intelligence, leadership style, organizational commitment, trust, job performance

## Abstract

This study examines the effects of leader emotional intelligence, leadership styles (transformational and transactional), organizational commitment, and trust on job performance. A questionnaire was administered to the participants, who were real estate brokers in Kaohsiung City. Of the 980 questionnaires administered, 348 valid responses were received, indicating an effective response rate of 35.5%. Structural equation modeling was used for the analysis. The results show that leader emotional intelligence has a significant and positive effect on trust in supervisors, and transformational leadership and trust within a team have significant and positive effects on job performance. In addition, organizational commitment has a significant and positive effect on job performance. Trust within a team mediates the significant and positive effect of leader emotional intelligence on job performance. Although transactional leadership has no direct, positive, and significant effects on employee job performance, trust in the workplace shaped by a leader’s leadership style will empower a team’s spirit and boost their morale, thereby indirectly affecting their job performance in a positive manner.

## Introduction

In today’s increasingly complex, ever-changing and fiercely competitive business environments, leaders must constantly enhance the competitiveness of their organization as well as their employees’ performance to survive and succeed. According to Masa’deh et al. (2016), employee job performance significantly and positively affects a company’s sales, because the sum of the tasks completed by every employee is reflected in the revenue of the company. Employees who are more capable of achieving set organizational goals contribute more to the company’s revenue. Therefore, it is crucial for organizations to implement measures that improve their employees’ job performance.

To sustain a company’s competitiveness, not only must supervisors motivate their employees to leverage their skills to achieve the company’s goals and interests, but it is equally important for a company to foster employees’ resonance with and loyalty toward organizational values. Organizational assets, such as important techniques or information, are obtained by employees as part of their job. Employees with a high level of organizational commitment identify themselves as a proud member of their organization who enjoys their job. Therefore, they are more efficient at work, have lower turnover intentions, are less likely to leak or steal confidential company information due to their loyalty, and avoid actions that damage their company’s reputation. In addition, employee–team-leader relationships are an indispensable component in business operations, especially regarding mutual trust. Team members who trust one another enhance their job performance through healthy interactions and teamwork (Jong and Elfring, 2010). Employees who comprehend the demands and work goals required by their supervisor and company trust their leader to reward them for their job performance. Employees who give timely and adequate assistance and support to their company in times of need generate trust toward their supervisor and company, which motivates them to work harder and achieve better performances (Schaubroeck et al., 2013).

Concerning the factors that affect job performance, in addition to trust and organizational commitment, it is also important to consider leader emotional intelligence, which affects trust and employee job performance. Emotional intelligence refers to an individual’s array of abilities to identify, express, understand, and evaluate their own emotions as well as those of others (Schlechter and Strauss, 2008; Harms and Credé, 2010). Kotsou et al. (2019) described emotional intelligence as the ability to identify, express, understand, manage, and use emotions. Emotional intelligence significantly affects our health, relationships, and work and learning experiences. Wen et al. (2019) proposed two ways to explain emotional intelligence. The first describes emotional intelligence as a social capability in which one is able to monitor their own as well as others’ emotions, develop emotional cognition-based information, and use this information to guide their thinking and actions. The second describes emotional intelligence as the capability to perceive one’s own and others’ emotions and initiate one’s emotional management and interpersonal relations. According to Wong and Law (2002), interpersonal interactions are essential for team operation; team members who are unable to regulate their emotions are more likely to find themselves at odds with others. Emotionally intelligent leaders who are aware of their subordinates’ emotional state help team members control their emotions and prevent conflicts between team members caused by overreactions (Chang et al., 2012; Liu et al., 2012). D’Errico et al. (2022) pointed out that leaders who possess humility and moral suasion facilitate a team to sustain their cooperative spirit and foster stronger within-team trust. Therefore, leader emotional intelligence does more than only allow employees to achieve better job performances, from an external viewpoint; it also stresses a leader’s humility and moral suasion, as well as controls the leader’s and their employees’ emotions, from an internal viewpoint. When both the internal and external criteria are achieved, successful teamwork and better job performance can be attained easily. Furthermore, emotionally intelligent leaders take the initiative to establish a positive and harmonious team morale and strengthen trust among team members, thereby enhancing the job performance and organizational effectiveness of managers and subordinates alike (Shahhosseini et al., 2012).

In addition, leadership styles are a factor that influences an employee’s trust in their supervisor as well as their job performance. Excellent leaders stimulate their employees to express their potential, actively enhance their job performance, and attend to their needs. On a psychological level, employees who resonate well with their leader’s leadership develop attitudes characterized by trust; on a behavioral level, they adopt behaviors that benefit their company and proactively dedicate themselves to achieving organizational goals. Therefore, organizational effectiveness is reflected through a leader, and leaders need to modify their leadership styles according to external environmental and temporal changes. Different leadership styles have different effects on organizational performance as well as the job performance of individual employees. Transformational leadership and transactional leadership are the two most common leadership styles. Transformational leadership stresses how leaders influence changes in their employees’ ideas and behaviors such that employees are committed to meeting organizational visions and goals. Yue et al. (2019) suggested that transformational leaders participate in goal setting and directly offer strategic directions for their organization while considering and meeting employee demands. They encourage employees to think from different perspectives and seize opportunities for personal growth. Transformational leaders provide instruction and training to help their employees adapt to new work environments seamlessly. These leaders motivate employees to fully engage and dedicate themselves to their job in order to improve their job performance (MacKenzie et al., 2001), thereby increasing their job satisfaction. The individualized support and motivation provided by transformational leaders make their employees feel like they are being cared for and not isolated or helpless. Subsequently, employees trust their leader’ leadership and are more willing to put more effort into their job and attain a better job performance (Braun et al., 2013). In transactional leadership, the leader–employee relationship is established on the grounds of transactions. Leaders set well-defined work goals and characteristics and implement rigorous reward or punishment schemes so that employees comprehend the benefits of carrying out their work tasks in accordance with their leader’s demands. Employees receive the rewards they desire by accomplishing their superior’s expected objectives. Transactional leaders need to set clear work standards for employees so they can understand the scope of their work and the basis for being rewarded or punished (Bass et al., 2003). These standards, in turn, generate employees’ trust in their leaders, causing employees to believe that high performers are rewarded whereas low performers or those who make errors are corrected or punished (Asencio and Mujkic, 2016). Jung and Avolio (2000) indicated that trust in supervisors mediates the indirect and positive effect of transformational leadership and transactional leadership on job performance. In the real estate industry, real estate brokers are classified as high-ranking brokers (high commission but no base salary) and typical brokers (low commission but high base salary). Therefore, branch managers who wish to understand the performance and development of each employee should leverage their own unique leader emotional intelligence (management of one’s and others’ emotions) to effectively formulate internal management mechanisms. To this end, transactional leadership should be applied when managing high-ranking brokers. Leaders should verify and define their subordinates’ roles, require them to achieve set goals, understand how the subordinates perform their roles to achieve specified results, and use performance as an ultimate indicator of achievement. Typical brokers place more emphasis on the individual differences between employees, and leaders affect the values and motivations of employees. In addition to motivating their subordinates to attain transactional objectives, the goal of leaders is to arouse their subordinates’ self-actualization, as opposed to immediately meeting self-interests.

Previous studies on job performance have mostly focused on employee satisfaction, organizational structure, and organizational commitment. For instance, Lee and Shen (2008) explored the effects of organizational structure, employee satisfaction, and organizational commitment on the job performance of real estate brokers. Hou (2012) examined the effects of organizational impartiality, organizational commitment, and trust on sugar company employees’ willingness to share knowledge as well as their job performance. Chang (2016) examined how organizational climate, organizational trust, leadership styles, and internal control affects employees’ organizational commitment and job performance. MacKenzie et al. (2001) discussed how job performance is affected by transformational leadership, transactional leadership, trust in supervisors, and role ambiguity. Schlechter and Strauss (2008) studied the relationships between leader emotional intelligence, transformational leadership, trust, and team commitment. The research framework of this study is based on those by MacKenzie et al. (2001), Schlechter and Strauss (2008), in which trust comprises trust in supervisors and trust within a team. The participants of this study are real estate brokers in Kaohsiung City. Structural equation modeling (SEM) was employed to explore the relationships and effects between leader emotional intelligence, trust, organizational commitment, transformational leadership, transactional leadership, and job performance.

Previous studies on the job performance of real estate brokers have mostly focused on how brokers’ task performance, job satisfaction, and loyalty affected their job performance (e.g., Abelson et al., 1990; Wang and Netemeyer, 2002; Lee and Shen, 2008; Acharya et al., 2010; Lee et al., 2017). To the best of the authors’ knowledge, few studies have collectively explored the effects of leader emotional intelligence, transformational leadership, and transactional leadership on job performance in the real estate brokerage industry. These three factors, however, have been examined for employees working in construction (Pryke et al., 2015), manufacturing (Schlechter and Strauss, 2008), and finance (MacKenzie et al., 2001; Walumbwa et al., 2008). Therefore, this study distinguishes itself by focusing on the real estate brokerage industry. The conceptual model in this study is established on three important constructs—leader emotional intelligence; transformational leadership; and transactional leadership, in conjunction with trust; organizational commitment; and job performance, whereby trust comprises trust in supervisors and trust within a team. Interestingly, this study employed three latent variables—leader emotional intelligence, transformational leadership, and transactional leadership, which are individually distinct in theoretical and definitional terms. The two leadership styles are conceptually different and are often included in model analysis [see Druskat (1994), Kalsoom et al. (2018), Purwanto et al. (2020)]; only one of these two mutually exclusive styles affects trust and job performance. The objectives of this study are as follows:

(1) To explore the effects of leader emotional intelligence, transformational leadership, transactional leadership, trust in supervisors, trust within a team, and organizational commitment on employee job performance.
(2) To examine whether leader emotional intelligence indirectly affects job performance through the mediator variables of trust within a team and transformational leadership.
(3) To examine whether transformational leadership and transactional leadership indirectly affect job performance through the mediator variable of trust in supervisors.

The participants of this study were real estate brokers in Kaohsiung City, Taiwan. According to the Real Estate Information Platform^1^ of the Ministry of the Interior, as of January 31, 2021, there are 659 registered real estate brokerage companies, Of these companies/offices, 659 operate normally, employing a total of 4377 real estate salespeople and 749 brokers (227 of whom double as land administration agents). Kaohsiung City ranks fifth (after Taichung, Taipei, New Taipei, and Taoyuan cities, respectively) among all cities and counties in Taiwan in terms of the number of real estate offices and employees, evidence of its considerable real estate industry size. In those 659 offices, each has two supervisors---a manager and a vice manager, which means there are at least 1318 supervisors in Kaohsiung City’s real estate brokerage industry. Furthermore, the remuneration scheme in the real estate brokerage industry differs from other industries as a broker’s income is determined from their commissions (brokers are ranked by their income into high, intermediate, and common).^2^ Moreover, because little prerequisite experience is required for newcomers, the brokerage industry has a particularly high turnover rate^3^ (Ministry of Labor, 2020).

Interestingly, business models in the real estate brokerage industry itself vary by business model (direct sales or franchise). Specifically, the selection of managers (leaders), remuneration scheme, employee management, and working hours differ considerably between the two business models. However, the focal points of this study are the effects of leader emotional intelligence, leadership style, organization commitment, and trust on employee job performance. SEM can be used to examine the differences between the coefficients of business models, selection of managers (leaders), remuneration scheme, employee management, and working hours, such as whether the effects of trust on job performance differ by business model. However, these examinations were not performed in the present study due to the sheer complexity of the analyses as well as being out of the scope of study. In addition, even if business models in the real estate brokerage industry itself vary by business model, then the selection of managers (leaders), remuneration scheme, employee management, and working hours will vary considerably. Job performance is a latent variable in this study and consists of task performance and contextual performance. The former refers to challenging work tasks and demands, work efficiency, and overall efficiency, which may differ systematically and therefore have no consistency in terms of task performance-related demands. For the sake of prudence, this study analyzed the differences between direct sales and franchise operations through cluster analysis in SEM. The resulting chi-square was greater than that of the non-clustered data, and the chi-square test for difference revealed that the non-clustered data had a better fit. On this statistical basis, it can be concluded that brokers in direct sales offices do not differ significantly in terms of estimated coefficients.

## Literature Review and Research Hypotheses

### Leader Emotional Intelligence, Transformational Leadership, Trust Within a Team, and Job Performance

Wong and Law (2002) suggested that emotionally intelligent and mature leaders exhibit higher awareness toward their own emotions as well as their subordinates’ and adopt mentally supportive actions that positively affect their subordinates’ job satisfaction and potential job performance. Pryke et al. (2015) found that in architectural team communications, project managers’ emotional intelligence (including their emotional sensitivity and emotional performance) has important impacts on the communication between managers and their subordinates. A project manager with excellent leadership is also one of the factors that contributes to a project’s success. Shahhosseini et al. (2012) studied the job performance of branch managers in the banking sector and found that the proper utilization of emotional intelligence increases the job performance and organizational effectiveness of managers and subordinates. Based on the above, this study proposes the following:

H1: Leader emotional intelligence has a significant and positive effect on job performance.

Bass (1985) identified four prerequisite behaviors among transformational leaders: intellectual stimulation, individualized consideration, idealized or charismatic influence, and inspirational motivation. Sosik and Megerian (1999) found that emotional intelligence is a promoter of transformational leadership. For instance, emotionally intelligent leaders have the ability to empathize with their employees and thereby exhibit individualized consideration to employees to overcome their difficulties at work. Employees who perceive their leader to be emotionally competent know that the leader is equipped with transformational leadership skills. Leaders who are able to perceive their own emotional competence and comprehend their influence over their subordinates enhance the effectiveness of their leadership (Day and Carroll, 2004). Transformational leaders and emotionally intelligent leaders share highly similar traits and behaviors (Barling et al., 2000; Prati et al., 2003). Leaders who exhibit excellent self-control over their emotions become a mainstay for their followers and increase the trust and respect of their followers. Leaders who are able to recognize the strengths, weaknesses, and traits of an employee allocate suitable tasks that empower the employee to help attain set organizational goals (Harms and Credé, 2010). Based on the arguments above, this study proposes Hypothesis 2 as follows:

H2: Leader emotional intelligence has a significant and positive effect on transformational leadership.

Podsakoff et al. (1990) proposed six key behaviors of transformational leaders: articulating a vision, providing a suitable work model for their subordinates, fostering the acceptance of team goals among employees, having high performance expectations, providing individualized support, and advocating innovation. MacKenzie et al. (2001) explored the leader behaviors of insurance salespeople and demonstrated that the individualized support provided by transactional leaders to their subordinates reflects the leaders’ respect for their subordinates’ opinions. These leaders also attend to their subordinates’ feelings and needs and assist in the development of their careers, thereby positively and significantly affecting their job performance. Walumbwa et al. (2008) focused on bank employees in the American Midwest and found that transformational leadership positively and significantly affects employee job performance via the mediator variables of enhancing employees’ self-efficacy and defining work goals. Liaw et al. (2010) showed that by enhancing employees’ customer orientation, transformational leadership positively affects employees’ service performance such that employees are willing to spend more time and effort to satisfy customers’ needs and successfully establish long-term service relationships with them. Noruzy et al. (2013) used SEM to explore the relations between transformational leadership, organizational learning, knowledge management, organizational innovation, and organizational performance in the Iranian manufacturing industry. The results reveal that transformational leadership has a positive effect on organizational performance and creates social environments that benefit the organization, thereby prompting subordinates to increase their job performance by engaging in higher-level knowledge management-related activities (such as organizational learning, organization management, and organizational innovation). Based on the above, this study proposes Hypothesis 3 as follows:

H3: Transformational leadership has a significant and positive effect on job performance.

Prati et al. (2003) found that emotionally intelligent teams build a higher level of trust, and leader emotional intelligence influences employees’ trust in their leader’s leadership. Schlechter and Strauss (2008) argued that emotionally intelligent leaders resolve conflicts through constructive approaches and establish human-centered cooperative relationships. In short, leader emotional intelligence has a significant and positive effect on trust among team members. Chang et al. (2012) noted that by helping team members control their emotions, emotionally intelligent leaders can prevent the negative impacts of employees’ overreactions and strengthen trust among team members. Liu et al. (2012) suggested that leader emotional intelligence, behaviors, and personalities are beneficial for fostering team morale and has positive effects on trust, communication, and participation among team members. Leaders also use their emotional intelligence to improve their trust in their team (Kauffmann and Carmi, 2014). On the basis of the above, this study proposes Hypothesis 4 as follows:

H4: Leader emotional intelligence has a significant and positive effect on trust in a team.

### Transformational Leadership, Transactional Leadership, Trust in Supervisors, and Job Performance

Yang and Mossholder (2010) suggested that employees’ trust in their leader is based on the leader’s perceived degree of impartiality and sincerity. Trust is conceptualized as employees’ belief in and loyalty to their leader. According to the study by MacKenzie et al. (2001), the core behaviors of a transformational leader (i.e., articulating a vision, providing a suitable work model for their subordinates, fostering the acceptance of team goals among employees, and providing individualized support) help employees believe that their leader will reward them based on their efforts. Therefore, transformational leadership has a positive and significant effect on trust in supervisors. Schlechter and Strauss (2008) highlighted that transformational leadership has a positive influence on trust in supervisors, because transformational leaders inspire their employees to achieve goals. Transformational leaders are also considerate of their employee’s well-being and motivate them to perform better, thereby increasing their trustworthiness among their employees. Jung and Avolio (2000) agreed that transformational leadership has a significant and positive effect on trust in supervisors. The authors suggested that throughout the process of achieving organizational goals, transformational leaders will exhibit their firm beliefs and altruism in order to motivate their employees to achieve organizational goals. Hence, when employees often highly regard their leader, they identify with the leader’s beliefs and have high respect toward them. Braun et al. (2013) studied the relationships between transformational leadership, trust within a team, trust in supervisors, and team performance among members in academic institutions. The results indicate that trust in supervisors mediates the positive and significant effect of transformational leadership on job satisfaction. Employees who trust their managers are more satisfied with their jobs because they are aware of the high level of concern and attention paid by their managers toward them (Yang and Mossholder, 2010). Weng and Li (2015) demonstrated that the behaviors of transformational leaders are manifested in their values, beliefs, and will. Team members who accept and internalize these traits naturally trust their supervisors. Asencio and Mujkic (2016) also found that transformational leadership has a significant and positive effect on trust in supervisors. Altunoğlu et al. (2019) found that a leader’s transformational leadership style, as perceived by employees, generates a positive impact on affective trust. Islam et al. (2021) agreed that transformational leadership has a positive effect on trust. Therefore, this study proposes Hypothesis 5, as follows:

H5: Transformational leadership has a significant and positive effect on trust in supervisors.

Employees who develop trust in their leader identify with their organization, which motivates them to work harder and achieve a better performance (Schaubroeck et al., 2013). In contrast, employees who lose trust in their leader are more likely to feel discontent. As a result of such negativity, employees feel more burnt out and have less enthusiasm in their work tasks (Bechtoldt et al., 2007). Jung and Avolio (2000) found that trust in leaders has a positive and significant effect on job performance. Dirks and Ferrin (2002) also agreed that trust in supervisors has positive effects on employees’ attitudes, behaviors, and job performance. Asencio and Mujkic (2016) highlighted that leaders should emphasize transformational leadership in order to strengthen interpersonal trust within their organization, motivate employees, and enhance organizational effectiveness. Mo and Shi (2017) studied employees working in a pharmaceutical retail company and found that trust in supervisors has a positive effect on employee job performance. Employees who distrust their leader lack the courage to communicate or express their feelings to them. Consequently, this results in a negative morale and climate within the organization. The literature above leads to the proposal of Hypothesis 6, as follows:

H6: Trust in supervisors has a significant and positive effect on job performance.

Nanjundeswaraswamy and Swamy (2014) observed that the relationship between transactional leaders and their subordinates is contract-based. Transactional leaders emphasize the needs and supervise the performance of their employees. They use reward and punishment schemes as a means for motivating employees to achieve organizational goals, i.e., employees are rewarded for their remarkable contributions to their company or punished if otherwise. Indeed, employees’ performance and future rewards are dependent on their job performance (Bass, 1985). As proposed by Franco and Matos (2015), transactional leadership is more effective when an organization encounters uncomplicated and definitive problems. In times of crisis, transactional leadership within an organization assists employees to focus on completing tasks that help overcome the crisis. Masa’deh et al. (2016) revealed that transactional leadership has significant and positive effects on job performance as it facilitates the enhancement of knowledge sharing within the organization and improves employees’ job performance. In their study on Malaysian private pharmacies, Basri et al. (2017) demonstrated that transactional leadership has significant and positive effects on job performance. Based on the findings of previous studies, this study proposes Hypothesis 7 as follows:

H7: Transactional leadership has a significant and positive effect on job performance.

Jung and Avolio (2000) indicated that transactional leaders gravitate toward gaining trust from their followers through contracts or exchanges of interests. Transactional leadership has a significant and positive effect on trust in supervisors. It also has an indirect and positive effect on job performance through the mediator variable of trust in supervisors. MacKenzie et al. (2001) highlighted how transactional leadership has a significant and positive effect on trust in supervisors. Transactional leaders who adopt contingent reward behaviors strengthen salespeople’s trust in supervisors as they believe managers will allocate rewards based on their sales performance. Asencio and Mujkic (2016) analyzed the leadership behaviors and trust in supervisors among American federal government employees using multivariate regression. The results indicate that employees trust the beliefs and behaviors of leaders who adopt transactional leadership behaviors as the employees believe that these leaders are able to distinguish between rewards and punishments, i.e., high-performing employees are rewarded whereas low-performing employees or those who made errors are corrected or punished. Greenberg (2003) also noted that in organizational management, leaders who are transactional gain the trust of their employees. On the basis of the above findings above, this study proposes Hypothesis 8 as follows:

H8: Transactional leadership has a significant and positive effect on trust in supervisors.

### Trust in Supervisors, Trust Within a Team, Organizational Commitment, and Job Performance

Jong and Elfring (2010) opined that organizational members who trust their teammates can reduce the suspicion and operational uncertainty within the team. Mutual trust between team members is established through good interactions and teamwork, and results in higher job performance. In contrast, team members who lack trust in one another tend to avoid interactions or cooperation to protect themselves from the actions of their teammates (Mayer and Gavin, 2005). This behavior ultimately has a negative impact on their performance as a team. Drescher et al. (2014) indicated that team members who build trusting relationships with one another are willing to put in extra effort to help their colleagues. As trust continues to build within the team, so does cooperation, thereby enhancing the team’s performance. Furthermore, mutual trust between team members reduces the time spent on supervising each other (Langfred, 2004). Ultimately, team members focus more on their work tasks (Serva et al., 2005). When more team members exhibit responsible and trustworthy behaviors, the team will invest more efforts in improving their workflow and enhancing their performance. Setiawan et al. (2016) argued that the attribute of trust positively affects an individual’s job performance. Varshney and Varshney (2017) agreed that trust has a positive effect on job performance. Based on the above, this study proposes Hypothesis 9 as follows:

H9: Trust within a team has a significant and positive effect on job performance.

Becker and Billings (1993) defined organizational commitment as the relative strength of an individual’s identification with, and engagement in, a specific organization. Organizational commitment consists of employees’ varying levels of commitment toward their supervisor, team, department, and organization as a whole. Cohen and Prusak (2001) demonstrated that employee job satisfaction and organizational commitment are established on the basis of trust. Schlechter and Strauss (2008) revealed that trust among team members has positive and significant effects on organizational commitment. Teams who build work relationships rooted in trust are able to strengthen their cooperation, reduce conflict, enhance organizational commitment, and decrease turnover intention. Yamaguchi (2013) agreed that trust within a team has significant and positive effects on employee job satisfaction and organizational commitment, and Chang et al. (2012) noted that trust has significant and positive effects on organizational commitment. Based on the above, this study proposes Hypothesis 10 as follows:

H10: Trust within a team has a significant and positive effect on organizational commitment.

A study by Jaramillo et al. (2005) on salespeople and non-salespeople demonstrated a positive relationship between organizational commitment and job performance. The relationship between the organizational commitment and job performance of salespeople is stronger than that of non-salespeople. Rose et al. (2009) found that organizational learning has a positive effect on job performance through the mediator variable of organizational commitment. Yeh and Hong (2012) highlighted the positive and significant effect of organizational commitment on job performance. Employees with intentions to stay within their organization dedicate themselves to completing their work goals based on their agreement with the organization’s values and goals. Therefore, leaders would give appropriate rewards when employees achieve their work goals. Supervisors who are able to achieve their own commitments lead to negativity among their subordinates, who become unwilling to put effort in their work and thereby lower their performance. Fu and Deshpande (2014) examined the relationships between caring climate, job satisfaction, organizational commitment, and job performance in a Chinese insurance company. The empirical results showed that organizational commitment had a significant and positive impact on job performance. Jamal (2011) investigated employees working at the Malaysian and Pakistani bases of a multinational company and found that organizational commitment had a significant and positive impact on job performance. In addition, work stress affected job performance through the moderator variable of organizational commitment. Based on the above, this study proposes Hypothesis 11 as follows:

H11: Organizational commitment has a significant and positive effect on job performance.

Lau and Moser (2008) highlighted the significant and positive effects of trust in supervisors on organizational commitment. Schlechter and Strauss (2008) agreed that trust in supervisors has a positive and significant effect on organizational commitment, and hence team leaders should develop organizational climates characterized by cooperation and trust. In so doing, leaders establish good organizational commitment and achieve effective teamwork. In their study on managers in the manufacturing and finance industries, Sholihin and Pike (2009) demonstrated the significant and positive effects of trust in supervisors on organizational commitment. Goh and Low (2013) examined the relationships between servant leadership, trust in leaders, and organizational commitment. The results indicate that trust in leaders has a positive and significant effect on organizational commitment. Trust in leaders is vital as it motivates employees to accept their leader’s beliefs and therefore facilitates the establishment of mutual cooperation between employees and leaders. The findings above lead to the proposal of Hypothesis 12 as follows:

H12: Trust in supervisors has a significant and positive effect on organizational commitment.

Regarding the mediating effects of the variables, briefly, this study also included several indirect and direct effects in its scope, including whether leader emotional intelligence indirectly affects job performance through the mediator variables of trust within a team and transformational leadership, as well as whether transformational leadership and transactional leadership indirectly affect job performance through the mediator variable of trust in supervisors.

## Research Design

### The Research Framework

**FIGURE 1:**
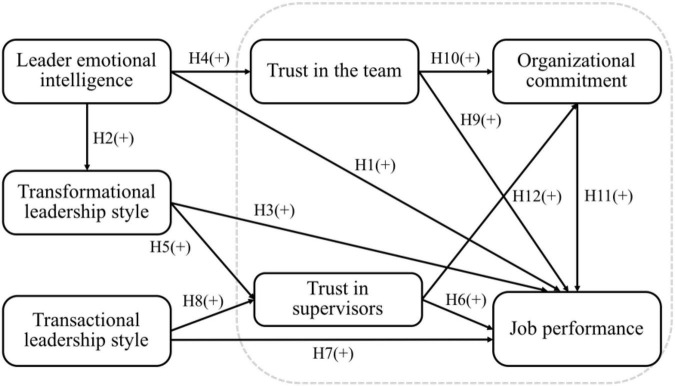
The research framework proposed in this study.

This research framework of this study, as shown in Figure 1, combines the three constructs of leader emotional intelligence, transformational leadership, and transactional leadership with a traditional job performance model that consists of trust within a team, trust in supervisors, organizational commitment, and job performance.

### Operational Definitions of the Variables

#### Leader Emotional Intelligence

Emotional intelligence refers to an individual’s ability to identify, express, understand, and evaluate their own emotions as well as those of others (Schlechter and Strauss, 2008). Davies et al. (1998) proposed a four-component model of emotional intelligence that consists of identifying one’s own emotions, identifying the emotions of others, utilizing emotions, and managing one’s own emotions. Identifying one’s own emotions refers to a behavioral agent’s observations and identification of their own emotions, which concerns their own subjective views of themselves. Since this study focuses on real estate brokers, the aforementioned component is excluded because how the emotional intelligence of leaders (store managers) affects their job performance as real estate brokers and how store manager perceive their own emotions do not fall within the scope of this study. This study refers to Davies et al.’s (1998) definition of emotional intelligence and suggests that the emotional intelligence of real estate brokers consists of the three dimensions of identifying others’ emotions, utilizing emotions, and managing one’s own emotions.

#### Transformational Leadership Style

Leaders with transformational leadership leverage their charm to motivate employees or meet their individual demands, thereby enhancing their job efficiency, confidence, and sense of authority, allowing them to surpass their own expectations and improve their job performance (Bass, 1985). Bass (1985), Bass and Avolio (1997) distinguished four traits of a transformational leader: idealized influence, inspirational motivation, intellectual stimulation, and individualized consideration. Podsakoff et al. (1990) proposed six key behaviors of transformational leaders: articulating a vision, assisting subordinates in finding suitable work models, fostering the acceptance of team goals among employees, having high performance expectations, providing individualized support, and advocating innovation. Following Bass’ (1985), Bass and Avolio’s (1997) definitions, the operational definition of transformational leadership in this study is a four-component model consisting of idealized influence, inspirational motivation, intellectual stimulation, and individualized consideration, whereby, in addition to evoking employees’ work motivations, supervisors encourage and express concern toward their employees promptly to help them achieve their work goals. Idealized influence refers to the ability of store managers to articulate organizational visions and influence employees’ job attitudes by establishing ambitious visions and objectives. Inspirational motivation refers to store managers’ behaviors in evoking employees’ job motivations, boosting their confidence, and enhancing their morale at work. Intellectual stimulation refers to store managers’ capabilities to encourage workers to examine the difficulties and predicaments they encounter at work, while also providing guidance and assistance to stimulate their problem-solving skills. Individualized consideration refers to the individualized assistance and support provided by store managers, as well as their respect for the demands and feelings of their employees.

#### Transactional Leadership Style

In the concept of transactional leadership, the leader–employee relationship is established on the basis of transactions. Leaders set well-defined work goals and standards and implement rigorous reward or punishment systems so that employees understand that they will be rewarded in return for achieving the objectives of the organization (Bass, 1985; Jung and Avolio, 2000). Bass and Avolio (1997) examined transactional leadership through contingent reward and management by exception. The former refers to the provision of individualized and materialistic rewards, while the latter refers to active and passive management models. This study refers to Bass and Avolio’s (1997) study and gives the operational definition of transactional leadership as leaders’ utilization of contingent rewards and management-by-exception measures to set well-defined work goals and standards, implement systems of reward or punishment and enhance employees’ job performance.

#### Trust Within a Team

Langfred (2004) explained that trust within a team is the aggregate perception of the level of trust among team members. With a high level of intra-team trust, team members focus more on their own tasks and less on monitoring one another. Staples and Webster (2008) defined trust within a team as the trusting relationships between coworkers and peers; that is, team members approve of each other’s job competence, acknowledge the mutual trust between them, and treat one another fairly. Jong and Elfring (2010) concluded that intra-team trust is the trusting relationships team members establish amongst themselves based on their beliefs in one another’s professionalism, dedication, work ethic, personality, and interpersonal relations. Based on the definition developed by Langfred (2004), Staples and Webster (2008), Jong and Elfring (2010), this study defines trust within a team as the level of trust between real estate brokers, and consisting of reliability, work ethic, impartiality, and interpersonal relations.

#### Trust in Supervisors

Lewis and Weigert (1985) explained trust in supervisors as employees believing in their supervisors’ leadership capabilities, ascertaining that the decisions made by their supervisors are beneficial for organizational developments, and perceiving supervisors as honest and considerate individuals that care about their employees’ demands and provide adequate assistance. MacKenzie et al. (2001) defined trust in supervisors as salespeople’s belief in their leaders’ impartiality and honesty. McAllister (1995) identified two types of trust: (1) Cognition-based trust, whereby a trustor perceives a trustee as trustworthy and competent based on their capabilities, expertise, or personalities (integrity, honesty, benevolence, and impartiality); (2) Affect-based trust, emphasizes the inclusion of emotional factors as the basis of trust between a trustor and a trustee, as well as the belief that the trustee will reciprocate these emotional efforts by showing the trustor their support, honesty, and care. This study adopts the cognitive theories of trust developed in the studies by McAllister (1995), MacKenzie et al. (2001) and defines trust in supervisors as the trusting relationship derived from a supervisor’s personality traits such as integrity, honesty, benevolence, impartiality, and faithfulness.

#### Organizational Commitment

This study measured several sub-dimensions of organizational commitment to achieve a deeper understanding of its implications. Mowday et al. (1982) developed a model of organizational commitment that consists of three sub-dimensions: (1) Propensity to identify with organizational goals, in terms of which an employee identifies with their organization by accepting the organizational goals and values and developing cohesion; (2) Willingness to contribute to the organization, which refers to an employee’s willingness to exert considerable effort for the organization; and (3) Tendency to retain in the organization, which refers to the desire to remain in the organization. Allen and Meyer (1990) developed an organizational commitment model with three components (affective, continuance, and normative commitment) as follows: (1) Affective commitment is generated from emotional attachment; (2) Continuance commitment is generated from perceiving the cost of leaving the organization; (3) Normative commitment is the sense of obligation to remain in the organization. This study adopts Allen and Meyer’s (1990) three-component model of organizational commitment to measure the commitment of real estate brokers to their branch offices.

#### Job Performance

Borman and Motowidlo (1993) described job performance as the aggregate of an individual’s behaviors in relation to organizational goals, and is measured by their contribution to these goals. Job performance is analyzed through task performance and contextual performance. The former refers to the outcomes of an employee’s expected or assigned tasks, quantitatively measured through indicators such as productivity and sales volume. The latter refers to the voluntary behaviors or performance of an employee, quantitatively expressed through the evaluations of supervisors and team members or the employee’s self-perceptions. Byars and Rue (1994) defined job performance as the extent to which an employee’s behavior contributes to organizational goals. The authors measured job performance based on the overall performance on the three dimensions of efficiency, effectiveness, and efficacy. Motowidlo and Van Scotter (1994) extended Borman and Motowidlo’s (1993) classification and definition of job performance, describing task performance as employees’ task outcomes measured by the extent to which they complete their organizational tasks while meeting the demands of their own tasks (job descriptions, standard operating procedures, and ad-hoc requests from supervisors). Contextual performance refers to an employee’s ability to perform prescribed activities that are not officially part of their work, enthusiasm in completing tasks, willingness to cooperate with and assist others, compliance with organizational regulations and procedures at their own cost, and support and defense of organizational goals. These behaviors are voluntarily expressed by employees and cannot be coerced by the organization. This study adopts the two sub-dimensions of task performance and conceptual performance delineated in Motowidlo and Van Scotter’s (1994) to measure the job performance of real estate brokers.

### Questionnaire Design

The questionnaire in this study consists of two sections. The first section consists of items pertaining to leader emotional intelligence, transformational leadership, transactional leadership, trust within a team, trust in supervisors, organizational commitment, and job performance. The items concerning leader emotional intelligence are developed on the basis of the study by Davies et al. (1998) and comprise three aspects—identifying emotions, utilizing emotions, and managing one’s own emotions. The items are revised according to the questionnaire by Law et al. (2004). Each aspect consists of two items, for a total of six items. The items concerning transformational leadership are developed on the basis of the studies by Bass (1985); Bass and Avolio (1997). There are four aspects—idealized influence, inspirational motivation, intellectual stimulation, and individualized consideration. The items are revised according to the questionnaire by Masa’deh et al. (2016). Each aspect consists of two items, for a total of eight items. The items concerning transactional leadership are developed on the basis of the study by Bass and Avolio (1997). There are two aspects—contingent reward and management by exception. The five items are revised according to the questionnaire by Masa’deh et al. (2016). The five items concerning trust within a team are designed according to the studies by Langfred (2004), Staples and Webster (2008), Jong and Elfring (2010). The five items concerning trust within a team are designed according to the studies by McAllister (1995), MacKenzie et al. (2001). Allen and Meyer (1990) classified organizational commitment into affective commitment, continuous commitment, and normative commitment. In this study, the items concerning organizational commitment cover these three aspects and are revised according to the studies by Meyer and Allen (1991), Fu and Deshpande (2014). Each aspect covers three items, for a total of nine items. The items concerning job performance are based on the study by Motowidlo and Van Scotter (1994), in which job performance consists of task performance and contextual performance. The questionnaire items are revised according to the studies by Fu and Deshpande (2014), Masa’deh et al. (2016). Each aspect consists of three items, for a total of six items. All the questionnaire items are measured on a five-point Likert scale (1 = strongly disagree; 2 = disagree; 3 = neutral; 4 = agree; 5 = strongly agree). The second section of the questionnaire covers the participants’ basic information, including their age, gender, tenure in the real estate brokerage industry, and annual income. The questionnaire in this study was designed alongside undergraduate students, who also used it as part of their graduation project. The questionnaire items are presented in Appendix 1.

### Sampling and Data Collection

The participants in this study consisted of real estate brokers employed at real estate franchises in Kaohsiung City. The real estate companies, with the number of branch offices in brackets, included Taiching Realty (59), Taiwan Real Estate (13), Yung-Ching Realty (43), H&B Housing (47), Chinatrust Real Estate Co. (19), Sinyi Realty (31), U-trust Housing (6), Pacific Realtor (2), ETWARM Real Estate Co., Ltd. (4), and Century 21 Real Estate (4). The geographical scope of research covered eight administrative districts in Kaohsiung City: Tsoying, Sanmin, Sanmin, Gushan, Lingya, Hsinhsing, Cianjin, Cianjhen, and Fongshan. These eight districts, which constituted the former Kaohsiung City (the former county-administered Kaohsiung City merged with Kaohsiung County to form a special municipality in 2010), were selected for their high density of real estate brokerage companies. The aforementioned 10 real estate brokerage companies listed a total of 224 branch offices on their websites. To include all the companies in the scope of research, each branch office was classified by location (administrative district) and then arranged by franchise. Subsequently, each branch office in each franchise was sampled, thereby ensuring that the sample covered all ten franchises in all eight districts. Ninety-eight branch offices were sampled in this study; 10 copies of the abovementioned questionnaire were delivered by the researchers in person to each branch office. Due to the nature of their work, the brokers had to be out of their offices at times, and hence the responses were collected three to five days after being administered. The survey period ran from May 1, 2018 to June 1, 2018. Of the 980 questionnaires administered, 411 were returned, of which 388 were valid, indicating an effective response rate of 39.59%. The expected sample size must be considered during sampling as it affects the accuracy of the estimation results. Assuming a tolerable error (d) of 0.05 and a level of significance (α) of 10%, this study requires a sample size (n) of 271 with a confidence level of 90%. This demand is met as there were 388 valid responses.

## Descriptive Statistics of the Sample

### Basic Participant Data

Among the valid responses, men accounted for 57.2% (199 people) of the participants and women accounted for 42.8% (149 people) of the participants. The mean age of the participants was 41 years. The eldest participant was 70 years old, and the youngest was 22 years old. Regarding marital status, married participants accounted for 51.1% (178 people), unmarried participants accounted for 44.0% (153 people), and participants with other marital statuses accounted for 4.3% (15 people). In terms of education level, university-level participants (including 4- and 2-year programs) accounted for the highest proportion of participants at 46.6% (162 people), followed by participants with high school (vocational) education or below, who accounted for 27.3% (95 people); participants with specialized education, who accounted for 20.1% (70 people); and finally, participants with master’s degrees and above, who accounted for 4.3% (15 people). In terms of job tenure, participants with 1 to 3 years of experience accounted for the highest proportion at 33.0% (115 people), followed by those with less than a year’s experience as well as those with 4 to 6 years of experience, who both accounted for 19.0% (66 people). In terms of company positions, participants in agent positions (brokers, salespeople) accounted for the highest proportion at approximately 91.7% (319 people), followed by branch managers, who accounted for 6.9% (24 people). In terms of company business model, franchises were the dominant model, accounting for approximately 81.0% (282 branch offices), whereas direct sales operations accounted for approximately 19.0% (66 branch offices). In terms of average annual income in the last 3 years, a majority of the participants (26.1%, 91 people) had an average annual income of between NT$310,000 and NT$500,000; followed by those with less than NT$300,000 (24.7%, 86 people); and then by those with an average annual income of between NT$510,000 and NT$700,000 (21.3%, 74 people).

### Data Processing

Before a questionnaire survey, it is important to consider the problem of non-response bias, which refers to any of the two following conditions which may result in an insufficiency of information obtained from the sample: (1) The responses are not collected in time or were returned only after the participants had to be reminded to do so. (2) There are missing data in the responses or the sample structure is too concentrated on a certain population or level. In addition, non-response bias also arises when representative samples for certain categories are missing in the questionnaire, which affects the completeness of the sample structure and thereby creates statistical bias (Chen and Wang, 2011). The questionnaire in this study was delivered by the researchers in person to each branch office, and the responses were collected 3 to 5 days later. Some of the responses were not returned in time, and the researchers had to remind the participants to return their responses. Therefore, the recovery process was completed twice, and a total of 348 valid responses were recovered. To ensure that the sample structure did not differ significantly between each recovery, as well as to ensure that the recovered data can be inferred to a population, the non-response bias test process proposed by Armstrong and Overton (1977) was employed to check for non-response bias in the sample. Firstly, the 162 responses returned in time were classified as Group 1, whereas the other 182 returned later were classified as Group 2. Next, the homogeneity test in the chi-square test (Armstrong and Overton, 1977) was used to check for homogeneity or consistency in the responses of both groups regarding the participants’ basic data (gender, age, marital status, education level, tenure, position, business model, and mean annual income). The results of the chi-square test are summarized in Table 1. All the *p*-values are greater than 0.05, and the null hypothesis is not rejected. This shows that there is consistency in the basic participant data of both groups. In other words, the non-response bias in the questionnaire survey is not significant, and the recovered data can be inferred to the population.

**TABLE 1:** Results of the chi-square test for non-response bias.

Item	Chi-square statistic	Degree of freedom	*p*-Value
Gender	0.898	1	0.343
Age	8.348	5	0.138
Marital status	0.701	2	0.704
Education level	2.244	3	0.523
Tenure	6.836	8	0.554
Position	0.674	2	0.714
Business model	0.122	1	0.727
Mean annual income	3.526	9	0.940

### Reliability and Validity Analysis

A reliability analysis checks the stability and reliability of a dataset, and is frequently measured using the Cronbach’s α. According to DeVellis (2016), a Cronbach’s α greater than 0.70 indicates that a scale has good consistency and stability. In this study, the Cronbach’s α of the seven latent variables ranged from 0.912 to 0.973 and were all greater than 0,70. Hence, the questionnaire designed in this study has a remarkably high reliability level (see Table 2).

**TABLE 2:** Cronbach’s α of each latent variable.

Variable	Cronbach’s α
Leader emotional intelligence	0.936
Transformational leadership	0.973
Transactional leadership	0.927
Trust within a team	0.927
Trust in supervisors	0.956
Organizational commitment	0.912
Job performance	0.941

Validity refers to the extent to which the measured variables of a scale are able to accurately measure the theme of a study (Chen, 2005). Validity consists of content validity, convergent validity, and discriminant validity. With regard to the content validity, the questionnaire in this study was designed after referring to and revising the questionnaire items used by relevant scholars from home and abroad. The questionnaire had to be in line with the scope of study and the research motivations. The items were developed, screened, and revised following the researchers’ discussions with real estate professionals. Therefore, the questionnaire should have a considerable level of content validity, and the convergent validity and discriminant validity are discussed in a subsequent section.

## Empirical Results and Discussion

The empirical results of this study are presented in terms of the measurement model and the structural model.

### Analysis of the Measurement Model

According to the recommendations of Nunnally and Bernstein (1994), DeVellis (2016), a construct has a high reliability if the Cronbach’s α is greater than 0.70, whereas a Cronbach’s α ranging from 0.50 to 0.70 is considered acceptable. As shown in Table 3, the Cronbach’s α of each variable is greater than 0.5, suggesting that the measured variables in this study have remarkable internal consistency and that the questionnaire results are within an acceptable range.

**TABLE 3:** Correlation matrix of latent variables.

	Leader emotional intelligence	Transformational leadership	Transactional leadership	Trust within a team	Trust in supervisors	Organizational commitment	Job performance
Leader emotional intelligence	0.903						
Transformational leadership	0.873	0.961					
Transactional leadership	0.849	0.741	0.948				
Trust within a team	0.830	0.725	0.705	0.911			
Trust in supervisors	0.788	0.780	0.818	0.654	0.950		
Organizational commitment	0.569	0.534	0.544	0.565	0.604	0.908	
Job performance	0.628	0.619	0.598	0.666	0.604	0.674	0.951

*The diagonal elements shown in this matrix are the square roots of the constructs’ AVE.*

This study employed SEM and used Anderson and Gerbing’s (1988) two-step approach for analysis.

Construct validity is measured in terms of factor loadings in this study. As shown in Table 3, the factor loading of each measured variable is statistically significant, demonstrating their excellent convergent validity. In addition, confirmatory factor analysis was performed to inspect the convergent validity and the discriminant validity of each construct. Regarding the evaluation of convergent validity, Anderson and Gerbing (1988) recommended that the measurement model of the structural model can be used to determine whether each measured variable can suitably measure each latent variable. As shown in Table 4, the factor loading of each measured variable is greater than 0.7 and statistically significant. Taken together, the questionnaire has an excellent convergent validity.

**TABLE 4:** Analysis of the questionnaire’s reliability, factor loading, and average variance extracted.

Variable	Factor loading (unstandardized)	Factor loading (standardized)	Error variance	Reliability of measured variable	Composite reliability (CR)	Average variance extracted (AVE)	Structural equation assessment *R*^2^
Leader emotional intelligence					0.930	0.816	
Identifying the emotions of others	1.000	0.839	0.170	0.704			
Utilizing emotions	0.980***	0.888***	0.105	0.789			
Managing one’s own emotions	0.924***	0.794***	0.203	0.631			
Transformational leadership					0.980	0.923	0.762
Idealized influence	0.974***	0.911***	0.087	0.830			
Inspirational motivation	1.013***	0.954***	0.045	0.911			
Intellectual stimulation	1.044***	0.927***	0.080	0.860			
Individualized consideration	1.000	0.924	0.077	0.853			
Transactional leadership					0.946	0.898	
Contingent reward	1.018***	0.911***	0.078	0.830			
Management by exception	1.000	0.883	0.105	0.779			
Trust within a team					0.961	0.830	0.689
Trust within a team 1	1.000	0.863	0.143	0.745			
Trust within a team 2	0.991***	0.855***	0.151	0.730			
Trust within a team 3	0.952***	0.846***	0.150	0.716			
Trust within a team 4	0.975***	0.886***	0.109	0.785			
Trust within a team 5	0.832***	0.784***	0.182	0.614			
Trust in supervisors					0.979	0.903	0.736
Trust in supervisors 1	0.992***	0.858***	0.138	0.736			
Trust in supervisors 2	0.937***	0.917***	0.065	0.840			
Trust in supervisors 3	0.986***	0.896***	0.093	0.803			
Trust in supervisors 4	1.002***	0.906***	0.086	0.821			
Trust in supervisors 5	1.000	0.935	0.056	0.874			
Organizational commitment					0.933	0.825	0.415
Affective commitment	1.000	0.700	0.211	0.490			
Continuous commitment	1.602***	0.924***	0.089	0.854			
Normative commitment	1.690***	0.890***	0.152	0.792			
Job performance					0.950	0.905	0.596
Task performance	0.932***	0.931***	0.054	0.867			
Contextual performance	1.000	0.879	0.119	0.773			

* denotes p < 0.1, ** denotes p < 0.05, *** denotes p < 0.01.

With regard to discriminant validity, Fornell and Larcker (1981) suggested that for a construct to discriminate well with other constructs, the square root of the average variance extracted (AVE) of a specified latent variable must be higher than the correlation coefficients of the other latent variables. For example, the correlation coefficient between leader emotional intelligence and transformational leadership is 0.873, which is smaller than the square root of their respective AVE (0.903 for leader emotional intelligence and 0.961 for transformational leadership). Therefore, based on analogous deductions, there is considerable discriminant validity between the constructs, as shown in Table 3.

### Analysis of the Structural Model

#### Evaluation of the Theoretical Model

The fit of the theoretical model was first tested using the chi-square statistic χ^2^(*p*-Value). If the value is statistically significant, then the theoretical model is not consistent with the data distribution structure, which means that other indices must be used instead. Common indices include the ratio of the chi-square statistic to the degree of freedom (χ^2^/*df*), the goodness of fit index (GFI), the root mean square residual (RMR), the root mean square error of approximation (RMSEA), the adjusted goodness of hit index (AGFI), the normal fit index (NFI), and the comparative fit index (CFI). Bagozzi and Yi (1988) proposed three approaches for measuring the fit of a model, i.e., preliminary fit criteria, fit of internal structure of the model, and overall model fit. Each approach is described as follows:

(1) Preliminary fit criteria:

According to Table 4, the factor loadings of the seven latent variables were all statistically significant and were greater than 0.7. There were also no negative values in the measured error variances. In general, the model of this study met the preliminary fit criteria.

(2) Fit of the internal structure of the model:

According to Bagozzi and Yi (1988), there are three approaches for evaluating the fit of the internal structure of a model, as follows: (1) The individual item reliability of each item is used to assess the construct reliability of measured variables to their corresponding latent variables, to validate whether a factor loading is greater than 0.5 and check the statistical significance of each loading. (2) The composite reliability (CR) of a latent variable is an aggregate of the reliabilities of its measured variables. It is used to check the consistency between the latent variables measured within a construct. Fornell and Larcker (1981) suggested that a CR greater than 0.6 indicates good reliability; the greater the CR, the higher the consistency of the internal construct indicators. The CRs of the latent variables in this study ranged were all greater than 0.9, reflecting the high consistency between the latent variables. (3) The AVE of a latent variable is the average percentage of variance of a measured variable that is explained by the latent variable (Chen, 2005). A high AVE indicates that the latent variable has a high convergent validity and reliability. Fornell and Larcker (1981) suggested that an AVE greater than 0.5 indicates good reliability. In this study, as shown in Table 4, the AVE values of the latent variables were all greater than 0.8, which indicates that the internal consistency of the questionnaire was acceptable.

(3) Overall fit of the model:

Bagozzi and Yi (1988) stressed that the fit of a structural model cannot be determined through a single indicator. Instead, the test results of the overall model reflect the fit of the structural model. Hair et al. (1998) noted that there are three types of overall model fit measures: absolute fit measures, incremental fit measures, and parsimonious fit measures. (1) Absolute fit measures determine the level of predictive covariates in the overall model. As shown in Table 5, the chi-square statistic (χ^2^) was 957.692 (*p* < 0.001) and was statistically significant. The null hypothesis is thus rejected, as the assumption model in this study did not have a good fit with the observed model. The chi-square statistic is very sensitive to sample sizes; a large sample size will increase the chi-square statistic, which results in the rejection of the null hypothesis. To resolve this issue, it is necessary to consider other fit indices for evaluating the fit of the overall model (Chiu, 2006). The values of the other indices are as follows: the normal chi-square statistic (χ^2^/*df*) was 4.007, which is smaller than 5; the RMR was 0.041; and the RMSEA was 0.093, which are both within an acceptable range; whereas the GFI was 0.818, which is close to 0.9. Generally speaking, the fit of the conceptual framework model is acceptable. (2) With regard to incremental fit measures, the AGFI was 0.771, which is close to 0.90; and the CFI was 0.922, which exceeds 0.90. Therefore, the incremental fit measures of the model are either acceptable or approximately acceptable. (3) Parsimonious fit measures, which are measured by adjusting the fit, determine the goodness of fit that can be obtained by each parameter estimate. The PNFI and PGFI in this study were 0.779 and 0.651, respectively, and were both greater than 0.50. This means that the parsimonious fit measures of the conceptual framework model devised in this study had a good fit. Furthermore, the values of all three fit measures attest to the good fit of the overall model.

**TABLE 5:** Goodness-of-fit indices of the conceptual framework model developed in this study.

Statistic		Benchmark for an ideal fit	Results
χ^2^ (*p*-value)		Not statistically significant	957.692 (0.001)
Absolute fit measures	χ^2^/*df*	Smaller than 5	4.007
	*GFI*	Greater than 0.90	0.818
	*RMR*	Favorably smaller	0.041
	*RMSEA*	Favorably smaller, ideal if smaller than 0.05	0.093
Incremental fit measures	*AGFI*	Greater than 0.90	0.771
	*NFI*	Greater than 0.90	0.900
	*CFI*	Greater than 0.90	0.922
Parsimonious fit measures	*PNFI*	Greater than 0.50	0.779
	*PGFI*	Greater than 0.50	0.651

#### Analysis of the Linear Structural Equation Model

**FIGURE 2:**
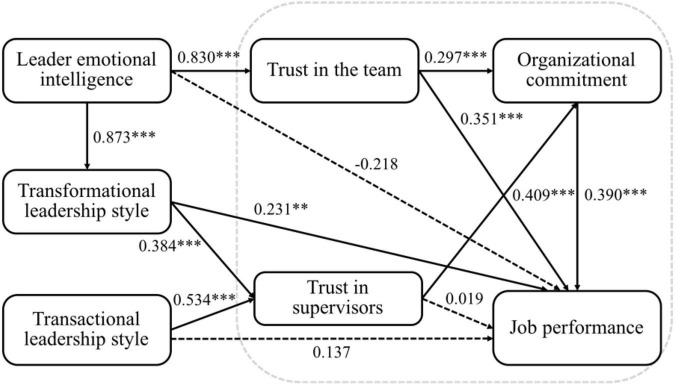
The linear structural equation model (with standardized coefficients). * denotes *p* < 0.1, ^**^ denotes *p* < 0.05, ^***^ denotes *p* < 0.01.

(1) Empirical results:

The empirical results of the linear structural equation model are presented in Figure 2 and Table 6 alongside the corresponding standardized estimated coefficients.

**TABLE 6:** Estimation results of linear structural equation modelling.

Hypothesis	Relationship between variables	Estimated coefficient	Standard error	t-statistic	*p*-Value	Outcome
H1	Leader emotional intelligence → Job performance	–0.218	0.181	–1.201	0.230	Not supported
H2	Leader emotional intelligence → Transformational leadership	0.873***	0.049	18.539	0.001***	Supported
H3	Transformational leadership → Job performance	0.231**	0.102	2.147	0.032**	Supported
H4	Leader emotional intelligence → Trust within a team	0.830***	0.053	15.918	0.001***	Supported
H5	Transformational leadership → Trust in supervisors	0.384***	0.050	7.165	0.001***	Supported
H6	Trust in supervisors → Job performance	0.019	0.092	0.212	0.832	Not supported
H7	Transactional leadership → Job performance	0.137	0.120	1.194	0.233	Not supported
H8	Transactional leadership → Trust in supervisors	0.534***	0.058	9.397	0.001***	Supported
H9	Trust within a team → Job performance	0.351***	0.089	3.874	0.001***	Supported
H10	Trust within a team → Organizational commitment	0.297***	0.046	4.486	0.001***	Supported
H11	Organizational commitment → Job performance	0.390***	0.086	6.403	0.001***	Supported
H12	Trust in supervisors → Organizational commitment	0.409***	0.048	6.088	0.001***	Supported

* denotes p < 0.1, ** denotes p < 0.05, *** denotes p < 0.01. All the estimated coefficients are standardized.

According to the results, the estimated coefficient of leader emotional intelligence on job performance was -0.218 but failed to reach the 10% significance level. This suggests that real estate brokers’ perceptions of their leaders’ emotional intelligence had no significant effect on their job performance; H1 is thus not supported. The estimated coefficient of leader emotional intelligence on transformational leadership was 0.873 with a 1% significance level; therefore, H2 is supported. The estimated coefficient of transformational leadership on job performance was 0.231 with a 5% significance level. This suggests that real estate brokers’ perceptions of their leaders’ transformational leadership had a significant and positive effect on their job performance; H3 is thus supported. The estimated coefficient of leader emotional intelligence on trust within a team was 0.830 with a 1% significance level, thereby demonstrating the positive and significant effect of leader emotional intelligence on trust within a team; therefore, H4 is supported. The estimated coefficient of transformational leadership on trust in supervisors was 0.348 with a 1% significance level, thereby supporting H5. The estimated coefficient of trust in supervisors on job performance was 0.019 but failed to reach the 10% significance level. This demonstrates that employees’ high level of perceived trust in their supervisor had no significant and positive effect on their job performance; hence, H6 is not supported.

The estimated coefficient of transactional leadership on job performance was 0.137 but failed to reach the 10% significance level. This demonstrates that employees’ perceptions of their leader’s transactional leadership behaviors had no significant and positive effect on their job performance; therefore, H7 is not supported. The estimated coefficient of transactional leadership on trust in supervisors was 0.534 with a 1% significance level. This suggests that employees who perceive that their leader exhibits the strong qualities of a transactional leader trust their leader even more; therefore, H8 is supported. The estimated coefficient of within-team trust on job performance was 0.351 with a 1% significance level. This shows that trust within a team has a significant and positive effect on job performance; hence, H9 is supported. The estimated coefficient of within-team trust on organizational commitment was 0.297 with a 1% significance level. This suggests that high levels of trust within a team strengthens employees’ perception of organizational commitment; H10 is thus supported. The estimated coefficient of organizational commitment on job performance was 0.390 with a 1% significance level; H11 is thus supported. The estimated coefficient of trust in supervisors on organizational commitment was 0.409 with a 1% significance level; therefore, H12 is supported.

The empirical results indicate that leader emotional intelligence had no significant and direct effects on job performance but had indirect effects through the mediator variables of within-team trust and transformational leadership. Transformational leadership had direct effects on job performance but had no indirect effects through the mediator variable of trust in supervisors. Finally, transactional leadership had no direct or indirect effects (through trust in supervisors) on job performance.

(2) Discussion:

This suggests that real estate brokers’ perceptions of their leaders’ emotional intelligence had no significant effects on their job performance, and thus H1 is not supported. An examination by Sy et al. (2006) on foodservice employees revealed that the estimated coefficient of leader emotional intelligence on job performance was 0.2, but this failed to reach the 5% significance level. The authors attributed this result to certain tasks, such as cooking, cleaning, and equipment maintenance, involving limited interactions with others. Consequently, there were no significant effects of leader emotional intelligence on job performance. Because half the participants in the study sample were newcomers with less work experience, they required more time to become acquainted with their leaders. Only when they had enjoyed a long professional relationship would subordinates perceive their leaders’ emotional intelligence. As such, this finding is similar to that of Sy et al. (2006). Wong and Law (2002) also found that leader emotional intelligence had no significant effects on job performance, which could be due to the participants’ profession as civil servants. Specifically, the requirement of civil service exams and the bureaucratic context could distort the performance ratings of subordinates. As a whole, the results of this study do not support H1, but H2 is supported. This finding is in agreement with the findings of Prati et al. (2003), Schlechter and Strauss (2008). Indeed, leader emotional intelligence has a positive and significant effect on transformational leadership. Sosik and Megerian (1999) observed that emotional intelligence promotes transformational leadership behavior. Emotionally intelligent leaders have the ability to empathize with their employees and exhibit individualized caring behaviors that help employees overcome their difficulties at work. Employees who perceive their leader to be emotionally competent know that leader is equipped with transformational leadership skills.

H3 is supported. Bacha (2014) explored the relationships between transformational leadership, job performance, and job characteristics among employees in the French service and manufacturing industries. The results demonstrate the significant and positive effect of transformational leadership on job performance. Manaf and Latif (2014) showed that through the mediating effect of the cultural trait of adaptability, transformational leadership had a significant and positive effect on job performance. Transformational leadership is rooted in having a clear vision and stimulating motivation. Transformational leaders are sometimes able to give pragmatic assistance to employees to boost sales, therefore generating significant effects on employee job performance.

H4 is supported. Employees’ strong perceptions of their leader’s emotional intelligence increase the level of trust within their team. This finding is in line with Liu et al. (2012), who found that emotionally intelligent leaders create positive team morale and build trusting relationships between members. When conflicts arise between team members, such leaders take appropriate measures to help them contain their emotions, resolve internal team conflicts, reduce the negative impacts of these conflicts, and enhance trust within the team (Chang et al., 2012). H5 is supported. This agrees with Asencio and Mujkic (2016), who found that transformational leadership has a positive and significant effect on trust in supervisors. Real estate brokers who perceive that their leader displays strong qualities of transformational leadership trust them even more. Schlechter and Strauss (2008) stated that transformational leaders help solve subordinates’ problems and recognize their efforts. In turn, subordinates trust in their supervisors is strengthened, and they are willing to dedicate themselves to the supervisor or the company. In the process of achieving organizational goals, transformational leaders display their firm beliefs and altruism as a means of motivating their employees to achieve organizational goals. Hence, when employees often regard their leader highly, they identify with that leader’s beliefs and have high respect for them (Jung and Avolio, 2000). In addition, transformational leaders are inspirational in the sense that they motivate employees to achieve goals while expressing concern for their well-being. Consequently, transformational leaders are perceived as trustworthy by their employees (Dirks and Ferrin, 2002). The core behaviors of a transformational leader, such as articulating a vision, providing a suitable work model for subordinates, fostering employees’ acceptance of team goals, and providing individualized support, encourage employees to believe that their leaders will reward their efforts and performance accordingly (MacKenzie et al., 2001). Weng and Li (2015) find that the behaviors of transformational leaders are reflected in their values, beliefs, and will. When team members accept and internalize these traits they tend to trust their supervisors. Taken together, transformational leadership has a positive and significant effect on trust in supervisors (Asencio and Mujkic, 2016).

H6 is not supported. Sheng et al. (2005) argued that interpersonal trust has negligible effects on employee job performance. Instead, this performance could be affected by other factors, including communication between members’ leadership styles, members’ task performance, and type of teamwork. Because half the study participants were newcomers with limited work experience, they required more time to become acquainted and build trust with their leaders. This scenario could explain the insignificant impact of trust in supervisors on employee job performance. The empirical results, however, show that even though trust in supervisors had no direct effects on employee job performance, there was an indirect effect through organizational commitment. In other words, brokers display their trust in their supervisor through organizational commitment. Even though broker’s trust in their supervisor did not have a direct and significant effect on their job performance, an indirect effect was generated through the mediator variable of organizational commitment.

H7 is not supported. Jung and Avolio (2000) indicated that whereas transactional leadership does not have a positive, direct, and significant effect on job performance, it does have a positive, indirect, and significant effect on job performance through the mediator variables of follower trust and value congruence. Shih (2006) concluded that transactional leadership had no significant effects on job performance because the remuneration scheme and standards of bank employees are fixed and well-defined. In this case, transactional leaders are unable to leverage their transactional traits to attain motivational effects. As the participants in this study are from the real estate brokerage industry, which has a defined set of duties and a clear remuneration scheme, transactional leaders may find their leadership skills have limited effectiveness within this context. H8 is supported. Asencio and Mujkic (2016) found that transactional leadership has a positive and significant effect on trust in supervisors. Transactional leaders are more likely to gain acceptance from their employees for their beliefs as the employees believe that such leaders are able to distinguish between rewards and punishments. In this regard, employees believe that they will receive the remuneration they deserve when they accomplish the goals set by their supervisor. Therefore, H8 is supported.

H9 is supported. Team members who trust their teammates can reduce suspicion and operational uncertainty within the team (Jong and Elfring, 2010). This mutual trust reduces the time members spend supervising one another (Langfred, 2004). Subsequently, team members focus more on their work tasks (Serva et al., 2005). Team members are willing to put in greater efforts to help their colleagues. As trust continues to build within the team, so does cooperation, thereby enhancing team performance (Drescher et al., 2014). Conversely, team members who distrust one another tend to avoid interaction or cooperation to protect themselves from the actions of their teammates (Mayer and Gavin, 2005). In real estate brokerage, a single customer may encounter different brokers throughout the different stages or forms of selling, such as product introduction, commissioned selling, and actual sale. Therefore, trust in their team is crucial for real estate brokers. Brokers who display responsible and trustworthy behavior during these different stages collectively improve their job performance. H10 is supported. This finding agrees with Yamaguchi (2013). Schlechter and Strauss (2008) argued that trust among team members contributes to better teamwork and organizational commitment. Geringer and Frayne (1993) likened the aspects of organizational commitment to construction materials; trust within a team is the concrete that firmly binds a brick wall. Strengthening trust within a team increases organizational commitment.

H11 is supported, and organizational commitment has a significant and positive effect on job performance. The results of this study are in line with Yeh and Hong (2012), Fu and Deshpande (2014). Indeed, organizational commitment has a significant and positive effect on job performance. Yeh and Hong (2012) concluded that employees who identify with their organization’s goals and values are willing to retain their positions and dedicate themselves to the organization.

H12 is supported. This finding is in line with that of Goh and Low (2013); that is, trust in supervisors has a significant and positive effect on organizational commitment. Schlechter and Strauss (2008) commented that leaders of organizations should establish team environments characterized by mutual trust and cooperation to enhance employees’ organizational commitment and loyalty and thus achieve effective team operations. MacKenzie et al. (2001) reported that trust in supervisors is the overall feeling of dependence displayed by employees toward their organizational leader. Organizational commitment is a series of behavioral expressions generated by organizational members on the basis of trust in their organization (Meyer and Allen, 1991). Employees who trust their organization have relatively higher organizational commitment. When employees have higher trust in their managers, organizational commitment is beneficial to the company’s sustainability (Sholihin and Pike, 2009).

## Conclusion and Recommendations

### Theoretical Implications

Focusing on real estate brokers and their leaders (store managers), this study explored the effects of leader emotional intelligence, transformational leadership, transactional leadership, trust, and organizational commitment on the brokers’ job performance. Leader emotional intelligence, transformational leadership, and transactional leadership constituted a traditional job performance model and served as important determinants of the job performance of real estate brokers. Trust in this study consisted of trust in supervisors and trust within a team, and the effects that both variables confer on job performance were also explored. Trust, commonly classified as individual trust of trust within a team, are both discussed in this study. In addition, leader emotional intelligence serves as an antecedent of trust within a team, whereas transformational leadership and transactional leadership serve as antecedents of trust in supervisors. In this regard, it is crucial to consider leader emotional intelligence, transformational leadership, and transactional leadership within analyzing trust in supervisors and trust within a team. Although previous studies have examined the relationships between leadership styles, emotional intelligence, and job performance individually, there is still a lack of comprehensive analyses on this topic. MacKenzie et al. (2001), Harms and Credé (2010), Bacha (2014), examined the effects of leadership style on job performance. Shahhosseini et al. (2012) examined the effects of emotional intelligence on job performance. Barling et al. (2000), Harms and Credé (2010) examined the association between leadership style and emotional intelligence. The lack of comprehensive analysis is addressed by this study examination of the relationships between leadership styles, emotional intelligence, and job performance by developing an integrated model of these variables on the basis of previous studies.

The empirical results indicate that leader emotional intelligence has positive and significant effects on transformational leadership and trust within a team, and transformational leadership has a positive and significant effect on trust in supervisors. In addition, transactional leadership has a positive and significant effect on trust in supervisors, and transformational leadership and trust within a team have positive and significant effects on employee job performance. Furthermore, trust within a team has a positive and significant effect on organizational commitment. However, leader emotional intelligence has no direct, positive, and significant effects on employee job performance, and trust in supervisors has no positive and significant effects on employee job performance. Moreover, transactional leadership also has no positive and significant effects on employee job performance. Even though the effects of leader emotional intelligence, trust in leaders, and transactional leadership on job performance do not support the hypotheses, the findings are consistent with real-life industry practices. This is due to the intense competition in the real estate industry, as it is easy for an individual to become a broker (Sinyi Realty, 2012).^4^ Most brokers earn their wages based on their sales performance, and prioritize the resources provided by their company (leader) to assist them in boosting their sales performance, while internal management-related factors such as leader emotional intelligence are secondary in importance.

Collectively speaking, even though leader emotional intelligence and transactional leadership have no direct, positive, and significant effects on employee job performance, trust in supervisors or the trust within a team as shaped by the leadership styles and emotional intelligence of leaders will boost a team’s spirit and morale, thereby affecting their job performance in an indirect fashion.

### Practical Implications

Firstly, leader emotional intelligence affects employee job performance through trust within a team, organizational commitment, and transformational leadership. In other words, emotionally intelligent leaders create a trustworthy and harmonious climate within a team, which helps improve subordinates’ job performance. Leader emotional intelligence includes leaders’ ability to control their own emotions, as well as recognizing and managing the emotional conflicts of others. Additionally, D’Errico (2019) studied the effects that arise from the emotional intelligence displayed (happiness, calmness, sadness, and anger) by political leaders when discussing ethical issues such as immigration. The results showed that humble politicians often elicit humility in their facial expressions and evaluations when communicating with others. This induces a state of personal distress toward immigrants among voters, especially when the politician displays a sad facial expression. Therefore, leader emotional intelligence does not imply a leader’s propensity to appease the public, but rather understanding their status and modifying strategies when appropriate.

Therefore, when training managers, real estate brokerage companies should stress the following actions: (1) Improving the observational skills of managers so that they can understand the needs of employees and customers, handle interpersonal conflicts effectively, and create harmonious and trusting work environments. (2) Strengthening the managers’ ability to control their emotions and handle affairs in a rational manner. (3) Enhancing the leaders’ emotional intelligence-related knowledge, which will help them resolve interpersonal conflicts in employees or customers.

Furthermore, transformational leadership has a direct effect on job performance and also has indirect effects through trust in supervisors and organizational commitment. When training managers, real estate brokerage companies should emphasize the development of leadership qualities in store managers so that they clearly understand their work goals and organizational visions, give timely motivations to employees, encourage innovation and self-improvement, take note of the opinions of their subordinates, satisfy employees’ demands, and provide necessary assistance. Moreover, leaders should actively build reliable work climates in order to boost employees’ loyalty and expectations toward their organization, thus increasing their willingness to dedicate themselves to the organization.

Lastly, transactional leadership affects job performance through trust in supervisors and organizational commitment. When training managers, real estate brokerage companies should emphasize the executive power of leaders by establishing well-defined work goals and content and appropriately implementing reward/punishment schemes. These measures will boost the discipline of team members as well as the authority of leaders. In addition, leaders should boost their integrity so that employees have faith and reliance in their organization, which generates a sense of belongingness to their organization. In this sense, they will believe that they will be rewarded for dedicating themselves to their job, thereby enhancing the employees’ performance and the operations of the organization.

### Limitations and Recommendations for Further Study

The questionnaire in this study was administered to real estate brokers, who completed all the items. It is suggested that the data could be obtained through more sources, such as the self-reported values of store managers and professionals. This enhances the understanding of the effects of leadership traits and styles on job performance. Next, further examination should be performed on the business models of the stores as well as the attributes of supervisors and employees alike. Finally, future studies can explore how other variables such as charismatic leadership, paternalistic leadership, knowledge sharing, personality traits, and corporate welfare affect the job performance of real estate brokers. Doing so would enhance the diversity and depth of the study.

In this study, SEM was used for analysis, which suffers from certain limitations, such as only being able to process the relationship between single-level variables, thus neglecting the effects of different variables. To overcome this issue, hierarchical linear modeling can be used for analysis, whereby variables are divided into the individual and aggregate levels, thus preventing the potential bias and underestimation that may arise in traditional statistical approaches (Courgeau, 2003). Going forward, future research can extend the factors in this study to analyze real estate properties at home and abroad. By examining the differences between various cultural backgrounds and environments (such as political environments), more information can be provided to facilitate the development and management of real estate business strategies.

## Data Availability Statement

The raw data supporting the conclusions of this article will be made available by the authors, without undue reservation.

## Author Contributions

All authors listed have made a substantial, direct, and intellectual contribution to the work, and approved it for publication.

## Conflict of Interest

The authors declare that the research was conducted in the absence of any commercial or financial relationships that could be construed as a potential conflict of interest.

## Footnotes

1 The Real Estate Information Platform of the Ministry of the Interior collates information on real estate appraisers, land administration agents, real estate brokerage, and rental and housing services. Website: https://resim.land.moi.gov.tw/Home/Queryindex. Last accessed on 2022.4.16.

2 High-ranking brokers do not have a base salary and rely solely on sales incentives; intermediate brokers have a low base salary and high sales incentives; and common brokers have a high base salary and low sales incentives.

3 Analysis on the Real Estate Industry, Ministry of Labor. Available at https://www.mol.gov.tw/media/1695/al01.pdf. Last accessed on 2020.2.20.

4 Source: Sinyi Realty (2012). Analysis of long-term investments. Website: https://statementdog.com/blog/archives/3225, Last accessed: 2022.3.30.

## Appendix

**APPENDIX 1:** The questionnaire items.

Construct	Item (Cronbach’s alpha)	Ref.
Leader emotional intelligence	Identifying the emotions of others	Davies et al., 1998; Law et al., 2004
	(1) I think the manager is strongly aware of an employee’s emotions. (0.726)	
	(2) I think the manager is able to identify an employee’s emotions by conversing with them. (0.696)	
	Utilizing emotions	
	(1) I think the manager is constantly setting up goals for employees and helping them to complete these goals. (0.719)	
	(2) When employees encounter a problem, the manager will always give them encouragement. (0.737)	
	Managing one’s own emotions	
	(1) I think the manager is able to control their own emotions and solve problems rationally. (0.701)	
	(2) I think the manager has a strong ability to control their own emotions. (0.683)	
Transformational leadership	Idealized influence	Bass, 1985; Bass and Avolio, 1997; Masa’deh et al., 2016
	(1) The manager will tell us their core values and beliefs. (0.789)	
	(2) I think the manager has a determined mindset. (0.812)	
	Inspirational motivation	
	(1) I think the manager will motivate me continuously. (0.855)	
	(2) The manager expresses their confidence and expectations in me. (0.839)	
	Intellectual stimulation	
	(1) The manager encourages me to express my own opinions and ideas. (0.820)	
	(2) The manager encourages me to ask and ponder thought-provoking questions. (0.797)	
	Individualized consideration	
	(1) I think the manager is considerate about the challenges I face in my work. (0.821)	
	(2) I think the manager is willing to go out of their way to guide their employees. (0.814)	
Transactional leadership	Contingent reward	Bass and Avolio, 1997; Masa’deh et al., 2016
	(1) When I perform well, the manager will give me positive feedback. (0.815)	
	(2) When I achieve a better sales performance, the manager will give me extra praise. (0.751)	
	(3) The manager will inform me of the rewards that I shall receive upon completing my tasks. (0.721)	
	Management by exception	
	(1) I feel that the manager delineates the shortcomings in my work. (0.739)	
	(2) I feel that the manager pays attention to employees who fail to achieve minimum sales. (0.723)	
Trust within a team	(1) I feel that my teammates and I can rely on each other. (0.682)	Langfred, 2004; Staples and Webster, 2008; Jong and Elfring, 2010
	(2) I feel that I trust most of my teammates. (0.669)	
	(3) I feel that my team is impartial. (0.726)	
	(4) I feel that my teammates will assist me when I encounter difficulties. (0.755)	
	(5) I feel great when I accomplish goals with my teammates. (0.704)	
Trust in supervisors	(1) I feel that the manager is impartial. (0.808)	McAllister, 1995; MacKenzie et al., 2001
	(2) I believe that the manager has excellent self-discipline. (0.791)	
	(3) I feel that the manager would not deceive their employees for their own benefit. (0.749)	
	(4) I feel that the manager keeps their word. (0.768)	
	(5) I believe that the manager has good motives and intentions. (0.793)	
Organizational commitment	Affective commitment	Meyer and Allen, 1991; Allen and Meyer, 1996; Fu and Deshpande, 2014
	(1) I enjoy sharing the status of my current company/branch office with other brokers. (0.506)	
	(2) I feel that I am a part of my current company/branch office. (0.683)	
	(3) My current company/branch office is of utmost significance to me. (0.745)	
	Continuous commitment	
	(1) It is not an easy task to leave my current company/branch office. (0.647)	
	(2) If I leave the company now, my lifestyle will change drastically. (0.559)	
	(3) I expect to stay at the current company/branch office for as long as I wish. (0.690)	
	Normative commitment	
	(1) I feel that the current company/branch office improves my livelihood. (0.721)	
	(2) To me, it is immoral to switch to another company/branch office. (0.608)	
	(3) Even if I have a better job, I would feel discontent after leaving my current company/branch office. (0.584)	
Job performance	Task performance	Motowidlo and Van Scotter, 1994; Fu and Deshpande, 2014; Masa’deh et al., 2016
	(1) I feel that I am able to overcome the difficulties in my work. (0.659)	
	(2) I take the initiative to solve problems. (0.720)	
	(3) I do not slack even when the manager is not in the office. (0.700)	
	Contextual performance	
	(1) I want to be assigned or handle challenging tasks. (0.668)	
	(2) On average, I have a considerably high work efficiency. (0.596)	
	(3) Generally speaking, I am able to complete the tasks demanded by the company. (0.684)	
